# Optimale internationale Arbeitsteilung

**DOI:** 10.1007/s10273-020-2632-4

**Published:** 2020-04-22

**Authors:** Thieß Petersen

**Affiliations:** Bertelsmann Stiftung, Carl-Bertelsmann-Straße 256, 33311 Gütersloh, Deutschland

**Keywords:** F10, F50, F60

## Abstract

Die internationale Arbeitsteilung bietet für eine Volkswirtschaft sowohl Vorals auch Nachteile. Sofern die damit verbundenen Grenzkosten und Grenznutzen die üblichen Verläufe aufweisen, gibt es für jedes Land ein optimales Ausmaß dieser Arbeitsteilung. Die Corona-Pandemie dürfte die Grundlagen für die entsprechenden Entscheidungen im Bereich der internationalen Arbeitsteilung–sowohl aus Sicht der Unternehmen als auch aus Sicht der gesamten Gesellschaft–deutlich verändern.

## Abstract (en)

The international division of labour implies both advantages and disadvantages to an economy. As long as the marginal costs and marginal benefi ts associated with the international division of labour stick to standard economic theory, there is an optimal degree of this division for every economy. The corona pandemic is likely to change the fundamentals for relevant decisions–both from the perspective of companies and of the society as a whole–in such a way that the extent of the division of labour and international trade will decrease correspondingly.

Die internationale Arbeitsteilung bringt eine Reihe von Vorteilen mit sich: Die Spezialisierung auf Güter, bei deren Produktion ein Land Kostenvorteile hat, fördert das Wirtschaftswachstum und steigert die Einkommen der Bürger. Die grenzüberschreitende Mobilität von Produktionsfaktoren sorgt für eine optimale Faktorallokation und bewirkt somit Produktivitäts- und Wachstumssteigerungen. Der internationale Wettbewerb fördert Innovationen und Produktivität. Schließlich erlaubt die Produktion für einen größeren Markt die Ausnutzung von Größenvorteilen mit entsprechenden Reduzierungen der Stückkosten und damit auch der Marktpreise.

Gleichzeitig sind mit der grenzüberschreitenden Arbeitsteilung und dem daran gekoppelten internationalen Handel auch Kosten verbunden: Der grenzüberscheitende Güterhandel erhöht die $CO_2$-Emissionen und den Ausstoß anderer Treibhausgase, was zu einer Beschleunigung des Klimawandels führt. Zudem erhöhen die längeren Transportwege die Transportkosten. Innerhalb der einzelnen Länder können sich soziale Spannungen ergeben, weil die Arbeitsteilung zwischen verschiedenen Ländern die Knappheitsverhältnisse der Produktionsfaktoren in jedem Land verändert und somit neben Gewinnern auch Verlierer hervorbringt. In entwickelten Volkswirtschaften sind letztere vor allem gering qualifizierte Arbeitskräfte (Autor, Dorn und Hanson, 2013).

## Theoretische Bestimmung des optimalen Ausmaßes der internationalen Arbeitsteilung

Eine Ausweitung der internationalen Arbeitsteilung (IA) ist für ein einzelnes Land ökonomisch betrachtet sinnvoll, solange der damit verbundene zusätzliche gesellschaftliche Nutzen (Grenznutzen = GN) größer ist als die zusätzlichen gesamtwirtschaftlichen Kosten (Grenzkosten = GK). Wird, wie in der volkswirtschaftlichen Analyse üblich, von steigenden Grenzkosten und einem sinkenden Grenznutzen ausgegangen, liegt das optimale Ausmaß der internationalen Arbeitsteilung in Abbildung 1 bei IA*.

## Bedeutung von Liberalisierung und Digitalisierung

Die Jahrzehnte nach dem Ende des Zweiten Weltkriegs waren davon geprägt, dass Handelshemmnisse – allen voran Zölle – weltweit abgebaut wurden. Damit verringerten sich die Kosten des grenzüberschreitenden Handels, was gleichzeitig eine Reduzierung der Kosten der internationalen Arbeitsteilung bedeutete. Der Abbau von Kapitalverkehrskontrollen und der Übergang zu flexiblen Wechselkursen hatten den gleichen Effekt, weil damit die finanzielle Abwicklung des internationalen Handels kostengünstiger wurde. Zudem senkte der technologische Fortschritt die Transport- und Kommunikationskosten. Das Ergebnis dieser Entwicklungen ist eine Verschiebung bzw. Drehung der Grenzkostenkurve in Abbildung 2 nach unten und folglich eine Ausdehnung des optimalen Ausmaßes der internationalen Arbeitsteilung von $IA_0^*$ auf $IA_1^*$.

**Dr. Thieß Petersen** ist Senior Advisor der Bertelsmann Stiftung in Gütersloh im Projekt „Global Economic Dynamics“ und Lehrbeauftragter an der Europa-Universität Viadrina in Frankfurt an der Oder.

Abbildung 1
**Bestimmung des gesamtwirtschaftlich optimalen Ausmaßes der internationalen Arbeitsteilung (IA*)**

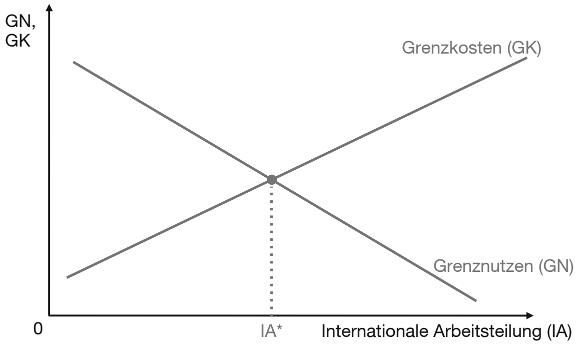

Quelle: eigene Darstellung.

Abbildung 2
**Wirkung von Liberalisierung (Lib.) und technologischem Fortschritt (TF)**

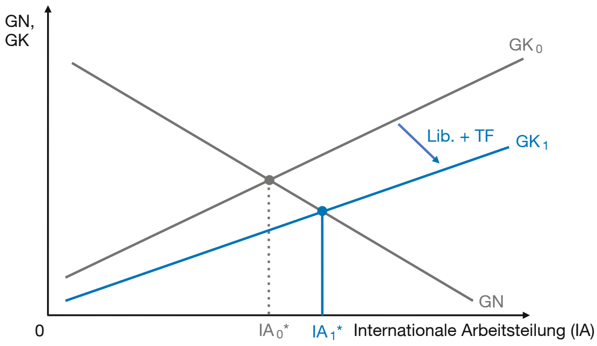

Quelle: eigene Darstellung.

Abbildung 3
**Wirkung eines negativen externen Effekts**

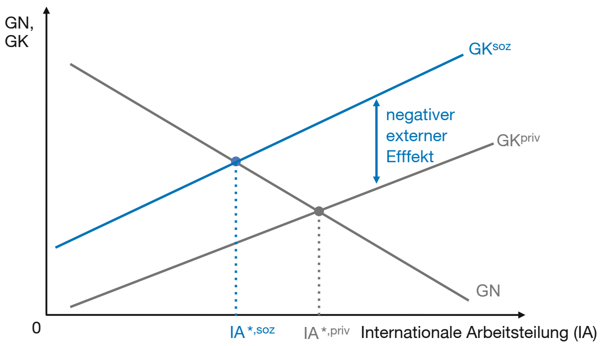

Quelle: eigene Darstellung.

## Auswirkungen einer Pandemie

Der Ausbruch der Corona-Pandemie hat für die Funktionsweise der internationalen Arbeitsteilung erhebliche Auswirkungen. Vor allem die zeitnahe Lieferung von aus dem Ausland stammenden Vorleistungen ist nicht mehr in jedem Fall gewährleistet. Die Störung der internationalen Lieferketten begann in Asien, wo mit China und Japan zwei der vier wichtigsten Zulieferer-Volkswirtschaften der Welt beheimatet sind (Baldwin und Tomiura 2020, 65 f.). Dort führte das krankheitsbedingte Fernbleiben von Arbeitskräften bzw. die Schließung von Produktionsstätten zu Produktionsausfällen. Sofern es für die asiatischen Vorprodukte in einem Importland wie Deutschland keine Substitute gibt, drohen in den betroffenen deutschen Unternehmen Produktionseinbußen oder sogar der komplette Stillstand der Produktion. Für die Unternehmen bedeutet das zusätzliche Kosten. Die Grenzkostenkurve wird somit in Abbildung 2 wieder ein wenig zurück nach oben gedreht (aus Gründen der Übersichtlichkeit nicht eingezeichnet), wodurch auch das optimale Ausmaß der internationalen Arbeitsteilung geringer wird. Praktisch würde das bedeuten, dass deutsche Unternehmen sich dazu entscheiden, besonders kritische Vorprodukte nicht mehr im Ausland herstellen zu lassen, sondern im Inland (Reshoring) – möglicherweise sogar im eigenen Unternehmen (Insourcing) (Heim, Matiz und Ehrat, 2014, 920). Auch die Bildung von größeren Lagerbeständen ist eine denkbare Handlungsoption, was jedoch die Lagerhaltungskosten steigen lässt.

## Bedeutung von negativen externen Effekten

Sofern alle Kosten in den Marktpreisen enthalten sind, sorgt der Markt – zumindest unter idealtypischen Rahmenbedingungen – dafür, dass das volkswirtschaftlich optimale Niveau der internationalen Arbeitsteilung erreicht wird. Problematisch wird es jedoch, wenn die Marktpreise nicht alle Kosten enthalten. In diesem Fall der negativen externen Effekte berücksichtigen private Wirtschaftsakteure nur die privaten Kosten, nicht aber die mit den externen Effekten verbundenen Kosten (also die gesamtwirtschaftlichen Zusatzkosten). Die Folge ist ein – gemessen am gesamtgesellschaftlich optimalen Niveau – zu hohes Ausmaß der internationalen Arbeitsteilung.

Zu den negativen externen Effekten, die mit einer Verlagerung einzelner Produktionsschritte in das Ausland verbunden sind, gehören z. B. die $CO_2$-Emissionen, die bei dem Transport von Gütern bzw. Vorleistungen entstehen. Die von ihnen verursachten Klima- und Umweltschäden sind normalerweise nicht in den Marktpreisen enthalten. Andere gesellschaftliche Zusatzkosten entstehen, wenn ein Unternehmen in Deutschland seine Produktion einstellen muss, weil die notwendigen Vorleistungen nicht mehr zur Verfügung stehen. Die Zusatzkosten fallen unter anderem in Form einer höheren (temporären oder dauerhaften) Arbeitslosigkeit sowie den damit verbundenen Transferzahlungen an. Wenn es sich bei den ausfallenden Importen um Vorleistungen handelt, die für überlebenswichtige medizinische Produkte benötigt werden, kann sich der negative externe Effekt auch in Form eines höheren Krankenstands oder sogar in Form von Todesfällen äußern.

In Abbildung 3 entspricht der negative externe Effekt, der mit der Ausweitung der internationalen Arbeitsteilung um eine infinitesimale Einheit verbunden ist, der Differenz zwischen den gesamtgesellschaftlichen Grenzkosten ($GK^{soz}$) und den privaten Grenzkosten ($GK^{priv}$). Die Entscheidungen von eigeninteressierten Individuen, die die gesellschaftlichen Zusatzkosten in ihrem Entscheidungskalkül nicht berücksichtigen, führen zu einem Ausmaß der internationalen Arbeitsteilung ($IA^{*,priv}$), das größer ist als das aus gesamtwirtschaftlicher Sicht optimale Niveau ($IA^{*,soz}$). Um das gesamtgesellschaftlich optimale Niveau zu realisieren, muss der Staat intervenieren, z. B. indem er den negativen externen Effekt mithilfe einer Steuer internalisiert.

## Ausblick

Die aktuelle Corona-Pandemie verdeutlicht die Anfälligkeit eines auf Lieferketten angewiesenen Produktionssystems. Der ungeplante Ausfall von Vorprodukten stellt für die betroffenen Unternehmen zusätzliche Kosten dar. Werden sie bei den Kosten-Nutzen-Analysen berücksichtigt, kann dies gegebenenfalls zu der betriebswirtschaftlich sinnvollen Entscheidung führen, auf einen Fremdbezug einiger wichtiger Vorprodukte zu verzichten, selbst wenn damit die Produktionskosten steigen. Das dann erreichte Ausmaß der internationalen Arbeitsteilungen – und damit auch des damit verbundenen grenzüberschreitenden Handels – dürfte somit als Reaktion auf die Erfahrungen während der Pandemie zurückgehen.

Ein partieller Verzicht auf die Vorteile der internationalen Arbeitsteilung hat unweigerlich höhere Kosten und damit auch höhere Preise zur Folge. Es kommt also zu einem realen Wohlstandsverlust. Dieser trifft einkommensschwache Haushalte härter als einkommensstarke. Damit stellt sich die sozialpolitische Frage, wie diese Kosten innerhalb der Gesellschaft verteilt werden. Dies gilt insbesondere, wenn lebensnotwendige Produkte betroffen sind. Wenn beispielsweise bestimmte medizinische Wirkstoffe zu höheren Kosten im Inland hergestellt werden, muss geklärt werden, wer diese zusätzlichen Kosten trägt: Steigen die Sozialversicherungsbeiträge (und wenn ja, bei wem: paritätisch bei Arbeitgebern und Arbeitnehmern oder nur bei Arbeitnehmern, um die internationale Wettbewerbsfähigkeit der deutschen Unternehmen nicht zu schwächen?) oder müssen die Patienten die Differenz selbst tragen oder erhalten die pharmazeutischen Unternehmen eine Subvention, sodass der Preis der Medikamente nicht steigt? Ein praktisches Umsetzungsproblem einer Relokalisierung der Produktion von Vorleistungen ergibt sich, wenn die Konzentration auf wenige ausländische Lieferanten in bestimmten Produktsegmenten und deren Lieferketten zu einer Monopolisierung geführt hat, weil dann schlichtweg keine anderen Anbieter zur Verfügung stehen (Helmold und Terry 2017, 168).

Selbst wenn es zu einer Verringerung der internationalen Arbeitsteilung und des damit verbundenen Handels kommt, bedeutet das keinesfalls das Ende der Globalisierung. Möglicherweise verlagern die Zulieferer Teile ihre Produktionsstandorte in die Länder, in die sie momentan noch exportieren. An die Stelle des grenzüberschreitenden Güterhandels treten dann ausländische Direktinvestitionen. Die internationale Vernetzung der Volkswirtschaften ändert sich in diesem Fall, aber sie geht nicht zurück.
